# Host Selection Behavior of the Green Peach Aphid, *Myzus persicae*, in Response to Volatile Organic Compounds and Nitrogen Contents of Cabbage Cultivars

**DOI:** 10.3389/fpls.2019.00079

**Published:** 2019-03-12

**Authors:** Nazeer Ahmed, Hewa Lunuwilage Chamila Darshanee, Imtiaz Ali Khan, Zhan-Feng Zhang, Tong-Xian Liu

**Affiliations:** ^1^State Key Laboratory of Crop Stress Biology for Arid Areas, Northwest A&F University, Yangling, China; ^2^Key Laboratory of Integrated Pest Management on the Loess Plateau of Ministry of Agriculture, Northwest A&F University, Yangling, China; ^3^Department of Agriculture, University of Swabi, Swabi, Pakistan; ^4^Graduate Research School, University of Southern Queensland, Toowoomba, QLD, Australia; ^5^Department of Entomology, The University of Agriculture, Peshawar, Pakistan

**Keywords:** cabbage cultivars, volatile blend, Y-tube olfactometer, aphid arrestment, nitrogen content

## Abstract

Plants emit volatile organic compounds (VOCs) in response to herbivore attack. VOCs emitted from the Chinese cabbage cultivars in response to the damage by the green peach aphid, *Myzus persicae*, were unknown. Using a solid-phase microextraction-based headspace collection method, we investigated and compared the emissions of VOCs from seven Chinese cabbage cultivars (Qibao, Qingan 80, Lvlong, Yuanbao, Qingan 70, Jinlv, and Lvqiu 66) in response to *M. persicae* attack. Our results showed that the VOCs emitted from the cultivars Qingan 80 and Yuanbao differed significantly from the other cultivars in response to the attraction of wingless *M. persicae*. Most importantly, out of the 27 detected compounds, α-caryophyllene was detected only in Qingan 80 and Qibao, but not in the other five cultivars. Among the compounds detected, 2 monoterpene and 12 terpenes were predominant in all cabbage cultivars. Furthermore, the wingless *M. persicae* showed preference to Qingan 80 while it had the highest nitrogen content among the tested cultivars. Moreover, we found a remarkable relationship among *M. persicae* attraction, plant nitrogen content, and total volatile emissions. Nitrogen content of the plants has a significant impact on volatile emission and preference behavior of *M. persicae.* Our results indicate that the wingless *M. persicae* were efficient in their interspecific host selection with an ability to distinguish plant cultivar differences by leaf nitrogen content. This study will be helpful in understanding aphid host selection, and sets a stage to further study the attractant-based integrated aphid management programs.

## Introduction

In natural conditions, plants produce a great diversity of volatile organic compounds (VOCs) during their metabolic activities. VOCs are released into the surrounding air and can be perceived by other organisms in the environment such as insects. VOCs are released by the reproductive structures like open flowers, pollen, buds and roots of the plants (Jonsson et al., 2005; Hiltpold and Turlings, 2012; Himanen et al., 2017). The mostly studied VOCs from plants for insect ecology are green leaf volatiles (GLVs: C9 and C6 fatty acid derivatives), terpenoids, amino acid derivatives like benzenoids, phenylpropanoids and some other compound like glucosinolates breakdown products from Brassicae (Pichersky and Gershenzon, 2002). The VOCs composition and magnitude of release changes with the plant parts, phenology, diurnal rhythm, and genotype (Dudareva et al., 2013). Plant VOCs are made and changed by different co-occurring biotic (e.g., infection by pathogens and feeding or oviposition by herbivores) and abiotic factors. The emitted volatiles reveal significant information about plant status and identity, since volatile emissions are different by the herbivore damages and also in plant cultivars within species (McDaniel et al., 2016). Specific groups of plant VOCs, such as isoprene, are released constitutively, whereas others are synthesized *de novo* (Kesselmeier and Staudt, 1999). The host plant selection behavior of herbivore pests can vary based on qualitative and quantitative changes of the plant VOCs. These VOCs can act as an attractant or a repellent for the herbivore (Verheggen et al., 2007, 2008; Dicke and Baldwin, 2010; Vandermoten et al., 2012). Insect can use VOCs to assess host quality, while also obtaining information about the plant’s natural enemies or competitors (Bengtsson et al., 2001; Magalhaes et al., 2012; Piesik et al., 2013). Several studies have shown that VOCs are produced by plants in response to an herbivorous insect’s attack, and these induced VOC emissions can confer protection through signaling compounds at various levels of the trophic network (Arimura et al., 2009). Moreover, plant-released VOCs comprise more than 200 different compounds, which could be used by phytophagous insects during their host selection and acceptance (Bruce et al., 2005; Webster et al., 2012).

Generalist herbivores take longer time compared to specialists because they have to make decisions among a wider selection of possible plants and therefore possible types of information (Troncoso et al., 2005). As a generalist herbivore the green peach aphid *M. persicae* (Sulzer) (Hemiptera: Aphididae) has a large host range and they use both visual and olfactory cues to locate their host plants (Pickett and Glinwood, 2007). The significant role played by olfaction in the pre- and post-landing stages of host selection varies depending on the species of aphid (Powell and Hardie, 2001). In general, aphids don’t have a lot of control over their movement but do seem to respond to plant color and odor. They can walk on an individual plant and do a little movement. (Webster et al., 2010), and distinguish the traits of their selected host plants based on individual VOCs or blends (Webster et al., 2010). Even as a generalist herbivore, *M. persicae* is capable of responding to host VOCs (Pickett and Glinwood, 2007) and distinguishing among the host VOC blends, including those elicited by viral infection (Eigenbrode et al., 2002; Ngumbi et al., 2007; Werner et al., 2009).

Herbivorous insects can be attracted or repelled by plants based on plant nutrient suitability and their experiences as well (Wang et al., 2008; Shah, 2017). Among plant nutrients, nitrogen contents of host plants play a significant role for preference and performance of herbivore pests. Some studies have observed changes of preference behavior of *M. persicae* (Jansson and Smilowitz, 1986) and the pea aphids (*Acyrthosiphon pisum* Harris) (Moravvej and Hatefi, 2008), due to variation in nitrogen concentration of their host plants. Further, plant volatile emission and herbivore attraction are related to the nitrogen content of plants (Olson et al., 2009). Therefore, we speculated that nitrogen levels of cabbage cultivars might affect volatile emissions that could alter attraction of *M. persicae*.

It is well known that interspecific variation in host plant suitability affects the survival and colonization of herbivorous insects in a natural system, which is one of the challenges involved in developing resistant cultivars (Fritz and Simms, 1992). In integrated pest management (IPM), the use of common pesticides can be minimized by practically applying semiochemicals (Shrivastava et al., 2010), trap crops or repellent cultivars (Birkett et al., 2004). The defensive and nutritional chemistry of the plant leaf is one of the factors that influences host choice and fitness of herbivore insects (Ahmed et al., 2018). Allelochemicals can influence selection of hosts by pest population (Slosser et al., 2004; Chau et al., 2005; Germano et al., 2011). On the basis of our previous study (Ahmed et al., 2018), in this study, we hypothesized that nitrogen content of cabbage cultivars affect the abundance of wingless *M. persicae*, and that this potential modification is related to changes in volatile compound of the plants. We tested our hypothesis by determination of nitrogen content in cabbage cultivars and observed host preferences of the wingless *M. persicae* through olfactory responses, and determined the plant volatiles emitted from cabbage cultivars. Finally, investigated whether nitrogen content in leaves was correlated with the attraction of green peach aphids and also plant volatile emission. Deciphering the VOC variation of cabbage cultivars and responses of wingless *M. persicae* will help to understand host selection of aphids.

## Materials and Methods

### Plant Materials

Seeds of seven commercially available cabbage (*Brassica oleracea*) cultivars used in our previous experiments (Ahmed et al., 2018); Jinlv and Lvlong (Xian Gong Si Seed Company Limited, Shaanxi, Liaoning, China), Lvqiu 66 (Nongcheng Seed Company Limited, Xian, Shaanxi, China), Qibao (Shaanxi Jinxing Seed Company Limited, Xian, Shaanxi, China), Qingan 70 (Kyoto Tolly Seed Company Limited, Kyoto, Japan), Qingan 80 (Yangling Nongcheng Seed Company Limited, Yangling, Shaanxi, China) and Yuanbao (Xiang Ganghuang Seed Company Limited, Hong Kong, China) were purchased from a local vegetable seeds market (Yangling, Shaanxi, China), and grown in the same environmental conditions (25 ± 2°C, 70 ± 10% RH and 12 h light: 12 h dark photoperiod) in 250-ml plastic pots containing a potting media (peat moss: perlite = 4:1) for 5 weeks. All plants were fertilized from a commercial fertilizer, “Kang Pu Jin” 20-20-20 of N-P_2_O_5_-K_2_O + Mg + TE (COMPO Expert GmbH, Krefeld, Germany), and watered as per necessary.

### Insects Rearing

The population of wingless *M. persicae* was obtained from a single wingless individual, which was reared on cabbage (*Brassica oleracea* “Qingan 50”) [Yangling Nongcheng Seed company Limited, Xian, China] in a greenhouse (25 ± 2°C, 70 ± 10% RH and 12 h light: 12 h dark photoperiod).

### Olfactory Choice Test With Y-Tube Olfactometer

A transparent glass Y-tube olfactometer that consisted of an 8-cm-long base with an internal diameter of 0.8 cm and two lateral 8-cm branches at an angle of 60° from each other was used to evaluate the behavioral response of wingless *M. persicae* to plant VOCs, following the previously described procedures of Akol et al. (2003) and Saad et al. (2015) with slight modifications. The two arms of the Y-tube individually connected to the two 3-L glass containers with odorless tubes, and each glass container individually contained the odor-loaded air from an intact cabbage plant of each cultivar. After the initial setup, the plant was kept in the glass container for 1 h before the bioassay to expel impure volatiles from the system. To minimize the effect of volatiles released from the potting mixture, the pot was first covered with ovenproof polythene fitted around the plant stem (Easy One Oven bags, Reynolds Kitchens, Lake Forest, IL, United States), and then with aluminum foil. Plants kept in the glass containers were covered by a piece of thick paper to ensure that *M. persicae* individuals received no visual cues from the plants. The charcoal-filtered humidified and purified air was provided at 120 ml min^−1^ to both branches of the Y-tube via odor sources using a vacuum pump (Beijing BCHP Analytical Technology Institute, Beijing, China) to circulate the VOCs. The airflow was adjusted and measured by an inline flow meter (LZB-3WB, Changzhou, Jiangsu, China). The adult *M. persicae* were starved for 2 h and then released within 0.5–1.0 cm of the base of the Y-tube with a small PCR tube, and their responses were assessed for 10 min. An aphid that walked half way or more toward the lateral branches of the Y-tube was considered as a responsive individual. If an individual made no decision within 10 min, it was considered as non-responsive insect. To eliminate differences in lighting across the Y-tube, a 20-W fluorescent light was placed 0.5 m above the Y-tube olfactometer. To vary the position of the two treatments, the two lateral branches of the Y-tube were rotated 180° after testing every five insects. Each olfactory test was repeated three times for each combination of stimulus pairs. Each replicate consisted of new 30 *M. persicae* adults and new set of plants. The bioassays were carried out between 12:00 and 16:00 h in a controlled environment maintained at 25 ± 1°C and 60 ± 5% RH. All glassware’s and Y-tubes were washed with soap and tap water, then rinsed with distilled water and finally sterilized with 70% ethyl alcohol before oven drying (120°C for 3 h) to reduce the contamination risk of the previously tested doors.

### Determination of Nitrogen Content in Cabbage Cultivars

Six cabbage plants (leaves with stem) were selected from each treatment and oven dried at 65°C for 4 days. The dried plant samples were ground with a mortar and a pestle, stored in a polythene bag and preserved at room temperature before analysis. Total nitrogen content in cabbage plant tissues was measured using the Kjeldahl method at FOSS Analytical (FOSS Innovation Centre, Hillerød, Denmark) was used to analyze total nitrogen content in cabbage plant tissues.

### Volatile Compound Collection in Cabbage Cultivars

Dynamic headspace volatiles of different cabbage cultivars were collected using the solid-phase micro-extraction (SPME) fiber coated with poly dimethyl siloxane-divinylbenzene (PDMS-DVB, 65 μm) purchased from Supelco (Bellefonte, PA, United States). Many studies have been carried out static headspace VOCs collection by SPME method. However, this method consequently increased the temperature and relative humidity in the chamber, which could affect the normal physiological processes involved in the emission of volatile chemicals (Gouinguené et al., 2001; Gouinguené and Turlings, 2002; Egigu et al., 2014). Therefore, in our experiments, VOCs were collected using a dynamic headspace SPME method. To minimize the volatiles emitted from potting mixture, the pot was covered with an ovenproof polythene bag fitted around the plant stem and then with a piece of aluminum foil. A large hole on top of the glass container lid was closed with a glass cover with a bend opening (4 mm in diameter), which was used to insert the SPME fiber. At the bottom of the glass container was another hole for ventilation. The SPME fiber was conditioned at 250°C for 30 min in a gas chromatograph injection port, according to the manufacturer’s guidelines. Plants were kept in the glass container for 1 h before sampling to eliminate any impure volatiles from the system. The SPME needle was inserted into the opening of the glass container, and the fiber was extended to absorb the plant volatiles that escaped in the dynamic headspace. After an hour, the SPME needle was inserted directly into a gas chromatograph-mass spectrometer (GC–MS) thermal desorption port, and thereafter the fiber was extended and kept for 5 min for the desorption of volatile substances. Each treatment repeated four times.

### Analysis of Volatile Compounds

The system consisted of a GC (TRACE 1310, Thermo Fisher Scientific, Waltham, MA, United States) that was used for the separation of volatile chemicals and an MS (ISQ Single Quadrupole MS, Thermo Fisher Scientific, Waltham, MA, United States) used for their detection, identification and quantification. The thermally desorbed VOCs were separated in a fused silica capillary column (30 m length × 0.25 mm diameter × 0.25 μm film thickness; Zebron, ZB-5 MS Ui). The programming split less injector temperature was maintained at 250°C, whereas the MS transfer line and ion source temperatures were maintained at 280°C. Purified helium (99.999%) was used as a carrier gas, and the flow rate was maintained constantly at 1.0 ml min^−1^. The initial GC oven temperature was set to 40°C for 4 min. The oven temperature was increased from 40 to 250°C at a rate of 8°C min^−1^ and held for 5 min. The MS was operated in electron ionization mode. The ion energy and emission current were maintained at 70 eV and 25 μA, respectively. The Xcalibur program (Ver. 2.1, Thermo Electron Corporation, United States) was used for data acquisition, which was performed in a total ion chromatogram with mass range from 33 to 500 amu. The identification of separated compounds was carried out with the NIST 2008 (National Institute of Standards and Technology, Washington DC, United States) database. The Kovats retention index of each volatile component was calculated using the retention time, and the data were matched to the previously published data (Table 1). The peak area of each component (tentatively identified) of the volatiles was the relative quantity.

**Table 1:** Volatile organic compounds identified in 7 cabbage cultivars and number responses of green peach aphid to 7 cabbage cultivars.

Peak No.	Compound	Cabbage cultivars						
		Qingan.70	Qibao	Qingan 80	Yuanbao	Lvlong	JinLv	Lvqiu 66
**Green leaf volatiles**								
1	*Trans*-2-Hexenal	ND	ND	5.64 ± 0.09	ND	ND	ND	ND
2	Hexanal	ND	ND	9.05 ± 0.22	ND	ND	ND	ND
3	(Z)-3-Hexen-1-ol,	8.02 ± 0.17	8.95 ± 0.20	9.42 ± 0.22	6.98 ± 0.12	7.67 ± 0.15	7.74 ± 0.15	7.51 ± 0.12
4	(Z)-3-hexenyl acetate	8.38 ± 0.14	8.93 ± 0.19	9.72 ± 0.24	3.75 ± 0.03	7.02 ± 0.19	6.48 ± 0.12	6.83 ± 0.12
**Terpenes**								
5	β-Thujene	3.24 ± 0.03	1.93 ± 0.01	9.11 ± 0.22	2.66 ± 0.02	6.19 ± 0.12	2.61 ± 0.02	6.63 ± 0.12
6	3-Carene	6.91 ± 0.12	ND	2.71 ± 0.01	1.43 ± 0.01	6.69 ± 0.13	3.97 ± 0.03	7.97 ± 0.15
7	á-Phellandrene	1.48 ± 0.01	5.80 ± 0.09	8.00 ± 0.17	ND	2.75 ± 0.02	2.05 ± 0.01	3.09 ± 0.03
8	á-Pinene	5.57 ± 0.07	5.95 ± 0.08	3.87 ± 0.03	2.80 ± 0.02	1.48 ± 0.01	1.32 ± 0.018	7.29 ± 0.15
9	à-Terpineol	1.29 ± 0.01	ND	ND	ND	2.24 ± 0.01	ND	ND
10	D-Limonene	1.37 ± 0.01	2.27 ± 0.01	1.30 ± 0.01	1.45 ± 0.01	4.23 ± 0.06	1.17 ± 0.01	4.25 ± 0.06
11	á-Ocimene	1.93 ± 0.01	5.84 ± 0.09	2.61 ± 0.02	9.47 ± 0.22	1.42 ± 0.01	1.00 ± 0.01	6.84 ± 0.12
12	*cis-*4-Thujanol	5.91 ± 0.09	1.09 ± 0.01	6.92 ± 0.02	1.66 ± 0.01	3.04 ± 0.03	2.73 ± 0.02	3.50 ± 0.03
13	(+)-2-Carene	ND	9.33 ± 0.22	ND	ND	ND	ND	ND
14	ç-Terpinene	3.02 ± 0.03	5.98 ± 0.09	6.28 ± 0.12	1.30 ± 0.01	1.90 ± 0.01	1.70 ± 0.01	3.76 ± 0.03
15	γ-Terpinene	5.91 ± 0.09	3.50 ± 0.03	9.44 ± 0.22	1.09 ± 0.01	2.73 ± 0.07	1.11 ± 0.01	3.04 ± 0.03
16	*Cis-*sabinene hydrate	3.60 ± 0.03	1.09 ± 0.01	1.12 ± 0.01	1.33 ± 0.01	1.48 ± 0.01	6.99 ± 0.12	6.05 ± 0.12
17	á-Terpinen-4-ol	1.59 ± 0.01	6.02 ± 0.12	7.17 ± 0.15	ND	1.54 ± 0.02	ND	2.08 ± 0.01
18	Caryophyllene	ND	1.49 ± 0.02	6.59 ± 0.12	ND	ND	ND	ND
19	*cis-*á-Farnesene	ND	ND	ND	5.88 ± 0.09	ND	ND	1.02 ± 0.01
20	Naphthalene	3.30 ± 0.03	3.30 ± 0.03	ND	ND	2.17 ± 0.01	ND	ND
21	α-Terpinonelene	2.08 ± 0.01	1.82 ± 0.01	4.97 ± 0.06	3.13 ± 0.03	1.02 ± 0.01	9.83 ± 0.22	2.54 ± 0.07
22	β-Myrcene	1.20 ± 0.01	9.79 ± 0.22	4.97 ± 0.06	1.18 ± 0.01	1.46 ± 0.01	1.88 ± 0.01	7.1 ± 0.15
23	(E)- β-farnesene	ND	ND	ND	6.76 ± 0.15	ND	ND	ND
24	1H-Phenalene	1.01 ± 0.01	ND	ND	ND	ND	ND	ND
25	(+)-4-Carene	2.44 ± 0.02	3.99 ± 0.03	ND	ND	ND	8.32 ± 0.17	1.70 ± 0.01
26	(+)-Camphene	7.87 ± 0.15	5.03 ± 0.09	2.27 ± 0.01	1.77 ± 0.01	5.67 ± 0.09	8.08 ± 0.17	6.90 ± 0.12
27	1,8-Cineole	1.21 ± 0.01	5.54 ± 0.09	7.66 ± 0.15	1.60 ± 0.01	5.82 ± 0.09	4.18 ± 0.06	0.0 ± 0.0
**Total**								
1	Green leaf volatile	16.40 ± 0.31	17.88 ± 0.39	26.84 ± 0.57	10.73 ± 0.15	14.60 ± 0.34	14.22 ± 0.27	14.34 ± 0.244
2	Terpenes	67.15 ± 1.43	72.17 ± 2.09	107.43 ± 2.61	40.02 ± 0.7	53.36 ± 1.18	60.23 ± 1.12	68.73 ± 1.66

*Values are mean ± SE (N = 4). ND indicates not detected.*

### Statistical Analyses

Multivariate analysis performed on relative abundance (i.e., compositional) data. The centered log-ratio transformation, mathematically expressed as:

Clr(x)=(log(x1/g(x)),……, log(xD/g(x)))

where x represents the composition, g(x) is the geometric mean of the composition x, and xD is Euclidean distances between the individual variables. We used the one-way analysis of variance (ANOVA), where means were separated by the least significant difference test. Bonferroni test was use for multiple testing, when comparing different plant cultivars. Data produced from Y-tube olfactometer choice bioassays were analyzed by *X*^2^ test. Cabbage plant volatiles and nitrogen contents were analyzed through one-way ANOVA; means were separated by the Tukey test. Correlation was used to identify a possible relationship between nitrogen, volatile constituent production, aphid preference of cabbage plants. On the basis of the relative abundance percentage of volatile compounds, principal component analysis (PCA) was used to determine whether plant cultivars in individual species belonged to separate groups. Nineteen major volatile compounds of cabbage cultivars were used to perform analysis. Volatiles produced in small amount were excluded from analysis. Score plots were used to visualize the results. In all cases, means were considered significant at *P* < 0.05 level. IBM SPSS statistics version 19 (Chicago, IL, United States) was used to conduct all statistical analyses.

## Results

### Olfactory Choice Test

**FIGURE 1:**
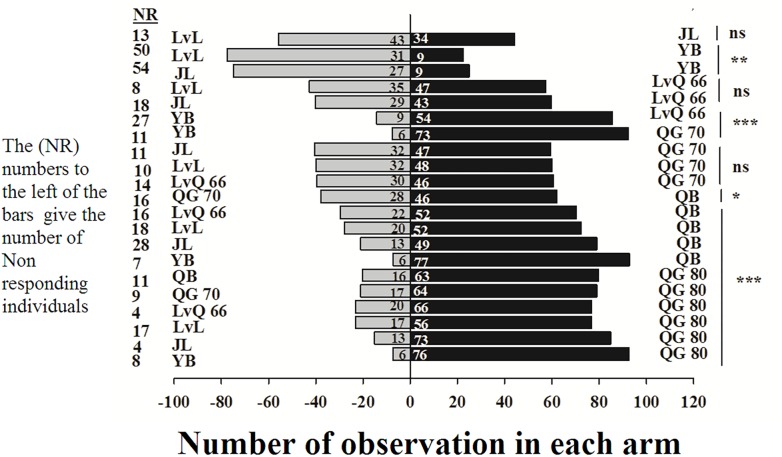
Number responses of green peach aphid in Y-tube olfactometer test were given an option between cabbage cultivar. NR, number of non-responsive insects; NS, non-significantly different at *P* > 0.05; ^∗^, significantly different at *P* < 0.05; ^∗∗^, significantly different at *P* < 0.01; ^∗∗∗^, significantly different at *P* < 0.001.

**FIGURE 2:**
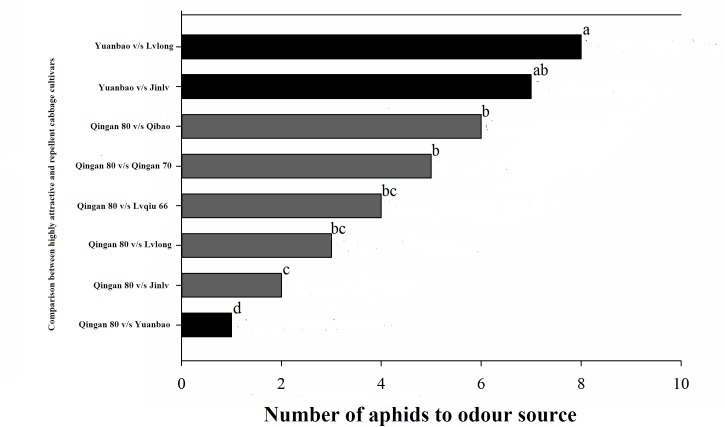
Number responses (NR) of green peach aphid in Y-tube olfactometer test were given an option between cabbage cultivar. (Highly susceptible, susceptible, resistant, moderate resistant, and highly resistant). a, ab, b, bc, c, and d letters showed significant differences among cabbage cultivars.

In the Y-tube olfactometer bioassays, the wingless *M. persicae* was significantly attracted to Qingan 80 compared with Yuanbao (χ^2^ = 59.75, *P <* 0.000), Lvlong (χ^2^ = 20.83, *P <* 0.000), Qingan 70 (χ^2^ = 27.27, *P* < 0.001), Qibao (χ^2^ = 27.96, *P* < 0.001), Lvqiu 66 (χ^2^ = 24.60, *P <* 0.001), and Jinlv (χ^2^ = 41.86, *P* < 0.001). Moreover, *M. persicae* selected Qibao when they relative to Yuanbao (χ^2^ = 60.73, *P* < 0.001), Jinlv (χ^2^ = 29.90, *P* < 0.001), Lvqiu 66 (χ^2^ = 12.16, *P* < 0.001), Qingan 70 (χ^2^ = 4.37, *P* < 0.036), and Lvlong (χ^2^ = 14.22, *P* < 0.001) (Figure 1). The preference of *M. persicae* was stronger on Qingan 70 than on Lvlong (χ^2^ = 3.20, *P* = 0.074), Jinlv (χ^2^ = 2.84, *P* = 0.091), Yuanbao (χ^2^ = 56.82, *P* < 0.001) or Lvqiu 66 (χ^2^ = 3.36, *P* = 0.066). Additionally, *M. persicae* showed preference to Lvqiu 66 over Yuanbao (χ^2^ = 32.14, *P* < 0.001), Lvqiu 66 over Jinlv (χ^2^ = 2.72, *P* = 0.099), Lvqiu 66 over Lvlong (χ^2^ = 1.75, *P* = 0.185), Jinlv over Yuanbao (χ^2^ = 9.00, *P* = 0.003), Lvlong over Yuanbao (χ^2^ = 12.10, *P* = 0.001), and Lvlong over Jinlv (χ^2^ = 1.05, *P* = 0.305). Among all tested cultivars, Qibao and Qingan 70 were observed as highly susceptible cultivars, whereas Yuanbao was the least preferred cultivar by *M. persicae*. In the Y-tube olfactometer bioassays, the response of wingless *M. persicae* was significantly different (*F*_7,56_ = 21.36, *P* = 0.012) when compared between highly repellent and attractive cultivars (Figure 2).

### Headspace Volatiles of Cabbage Cultivars

**FIGURE 3:**
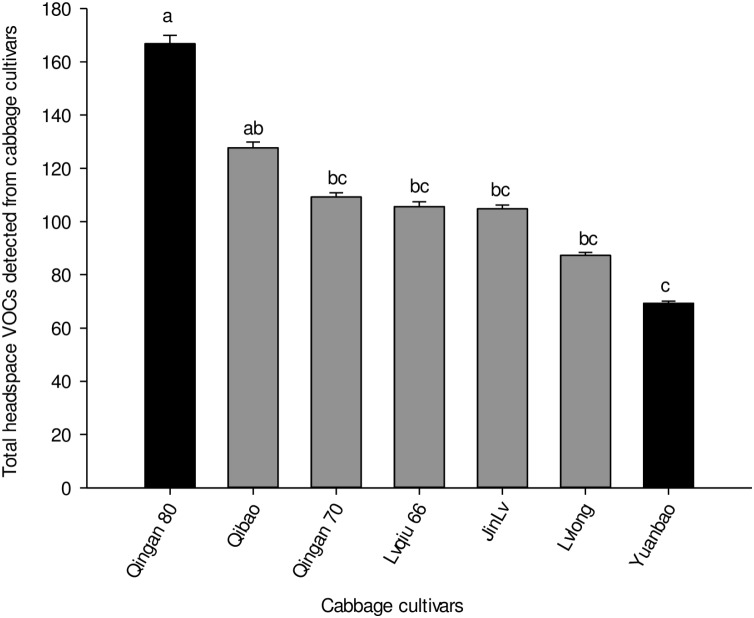
Total amounts of volatile organic compounds, terpenes, and green leaf volatiles detected in the seven cabbage cultivars. Different letters showed significant difference among cabbage cultivars.

Twenty-seven different chemical compounds, including 4 GLVs and 23 terpenes, were detected in seven cabbage cultivars. However, 19 compounds out of 27 showed statistical different among seven cabbage cultivars (*F*_6,28_ = 5.120, *P* = 0.001; Table 1). The highest amount of volatile emission was detected in Qingan 80 and Qibao, whereas the lowest was identified in Yuanbao. The total amount of volatiles in Qingan 80 and Qibao were higher than Yuanbao (*F*_6,28_ = 6.28, *P* = 0.003), whereas the remaining cultivars did not show significant differences with each other. The quantity of GLVs (*F*_6,2_ = 5.31, *P* = 0.005) and terpenes (*F*_6,42_ = 19.26, *P* = 0.004) detected in Qingan 80 and Qibao were significantly higher than those detected in Yuanbao (Figure 3).

There was nineteen volatile compounds showed significant different among seven cabbage cultivars such as hexanal (*F*_6,42_ = 7.92; *P* < 0.001), *Trans-*2-hexanal (*F*_6,42_ = 3.36; *P* < 0.001), α-pinene (*F*_6,42_ = 9.07; *P* < 0.001), β-thujene (*F*_6,42_ = 7.86; *P* < 0.001), 3-carene (*F*_6,42_ = 22.14; *P* < 0.001), á-ocimene (*F*_6,42_ = 22.20; *P* < 0.000), α-caryophyllene (*F*_6,42_ = 6.37; *P* < 0.001), α-terpinonelene (*F*_6,42_ = 4.27; *P* < 0.006), β-myrcene (*F*_6,42_ = 3.19; *P* = 0.02), (E)-β-farnesene (*F*_6,42_ = 7.84; *P* < 0.001), (+)-2-carene (*F*_6,42_ = 6.81; *P* < 0.001), (+)-4-carene (*F*_6,42_ = 14.41; *P* < 0.001), (+)-camphene (*F*_6,42_ = 4.33; *P* < 0.005), 1,8-cineole (*F*_6,42_ = 10.38; *P* = 0.003), *cis-*á-farnesene (*F*_6,42_ = 6.67; *P* < 0.001), á-terpinen-4-ol (*F* = 13.88; *P* < 0.001), γ-terpinene (*F* = 5.94; *P* < 0.001), ç-terpinene (*F*_6,42_ = 17.61; *P* = 0.002) and *cis-*4-thujanol (*F*_6,42_ = 3.02; *P* < 0.028).

**FIGURE 4:**
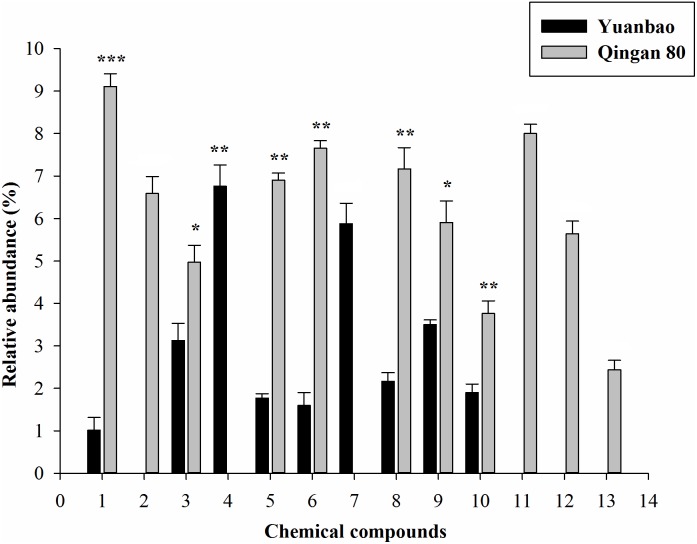
Quantitative difference of the identified volatile compounds emitted by seven cabbage cultivars. Each bar shows the relative amounts of (Mean ± SE) collected headspace from seven plants. ^∗^, significantly different at *P* ≤ 0.05; ^∗∗^, significantly different at *P* ≤ 0.01; ^∗∗∗^, significantly different at *P* ≤ 0.001.

Furthermore, α-ocimene (*F*_6,42_ = 10.61, *P* = 0.017), Thujanol (*F*_6,42_ = 1.62, *P* = 0.025), β-thujene (*F*_6,42_ = 10.23, *P* = 0.019), α-caryophyllene (*F*_6,42_ = 19.69, *P* < 0.001), *Trans-*2-hexenal (*F*_6,42_ = 10.41, *P* < 0.001), (E)-β-farnesene (*F*_6,42_ = 19.27, *P* < 0.001), Hexanal (*F*_6,42_ = 4.45, *P* = 0.001), 1,8-cineole (*F*_6,42_ = 18.78, *P* = 0.002), α-terpinen-4-ol (*F*_6,42_ = 12.58, *P* = 0.003), *cis-*α-farnesene (*F*_6,42_ = 2.04, *P* = 0.004), γ-terpinene (*F*_6,42_ = 11.19, *P* = 0.015), ç-terpinene (*F*_6,42_ = 17.02, *P* = 0.017) and α-phellandrene (*F*_6,42_ = 14.23, *P* < 0.001) had significant difference between the highest resistant and the highest susceptible cultivars (Figure 4).

**FIGURE 5:**
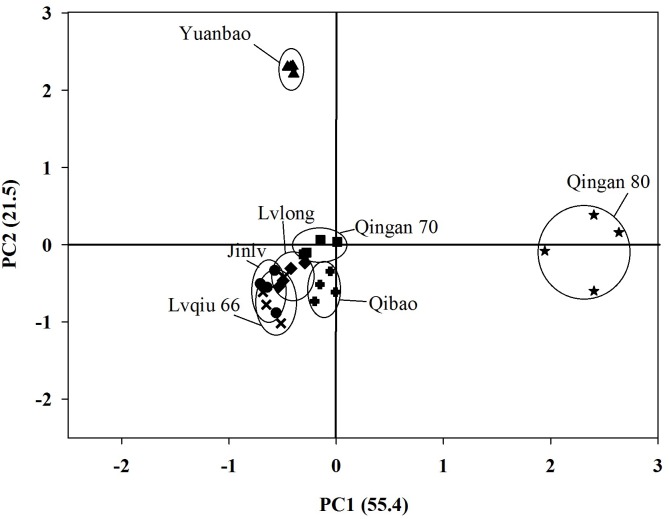
Principal Component Analysis (PCA) for seven cabbage cultivars based on the 16 different chemical compounds (measured as relative abundances of total peak area in individual cultivar in SPME headspace volatile collection.

A PCA for volatiles emitted by cabbage cultivars showed a clear separation among cultivars. First principal component separated Qingan 80 from the other six remaining cultivars and explained55.4% of the variance. Subsequently, Yuanbao was separated due to differences in volatiles from Qibao, Qingan 70, Lvqiu 66, Lvlong, and Jinlv at the second component (21.5%) (Figure 5).

### Nitrogen Content in the Leaves of Cabbage Cultivars

**FIGURE 6:**
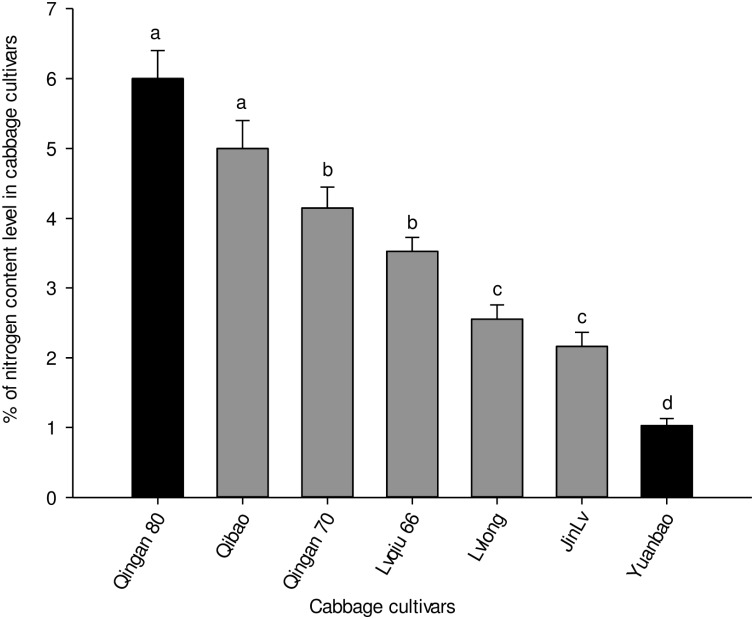
Percentages of total nitrogen (Mean ± SE) in cabbage plant tissues. Different letters showed significant difference among cabbage cultivars.

Significant differences were recorded in the nitrogen content of the leaves of seven cabbage cultivars (*F*_6,35_ = 7.173, *P* = 0.004). The highest nitrogen content was recorded in Qingan 80 and Qibao, whereas the lowest was observed in Yuanbao. The pattern of nitrogen content in the leaves of cultivars, from the lowest to the highestwas as follows: Yuanbao < Jinlv < Lvlong < Lvqiu 66 < Qingan 70 < Qibao < Qingan 80 (Figure 6).

### Correlation of Plant Nitrogen Content and Wingless *M. persicae* Attraction

**FIGURE 7:**
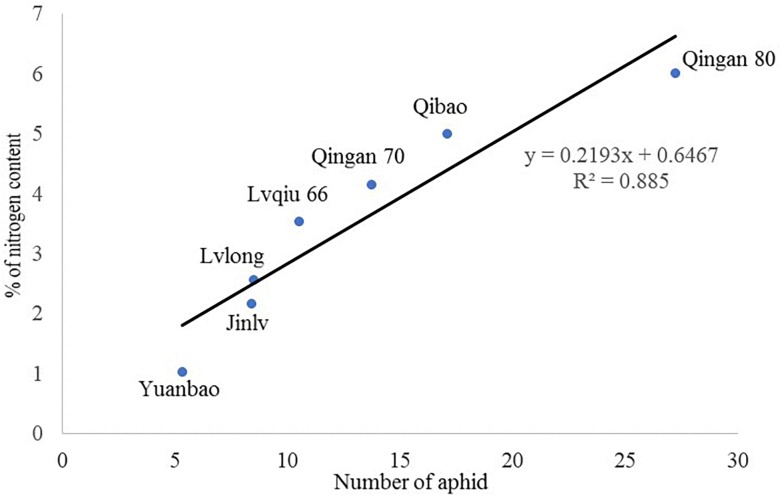
Correlation of nitrogen content with wingless *M. persicae.*

A significant (*P* = 0.002) and a positive (*r* = 0.885) correlation was observed between wingless *M. persicae* population and nitrogen content of cabbage cultivars. *M. persicae* were attracted to the cultivars having higher nitrogen content in the leaves (Figure 7).

### Correlation of VOCs With Nitrogen Content

**FIGURE 8:**
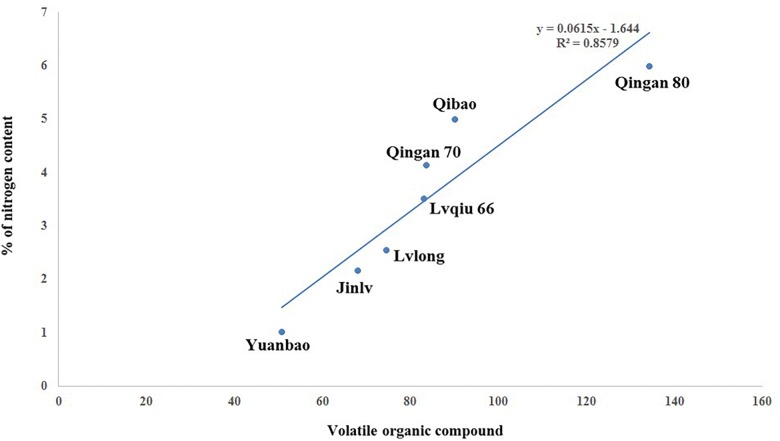
Correlation of VOC with wingless *M. persicae.*

A significant (*P* = 0.001) and positive (*r* = 0.858) correlation was observed between wingless *M. persicae* and VOCs among cabbage cultivars. As the VOCs increased, the aphid preference also increased. Moreover, *M. persicae* are more attracted to cultivars with higher nitrogen content that release higher amount of VOCs (Figure 8).

### Correlation of VOCs, Nitrogen Content and Aphid

**FIGURE 9:**
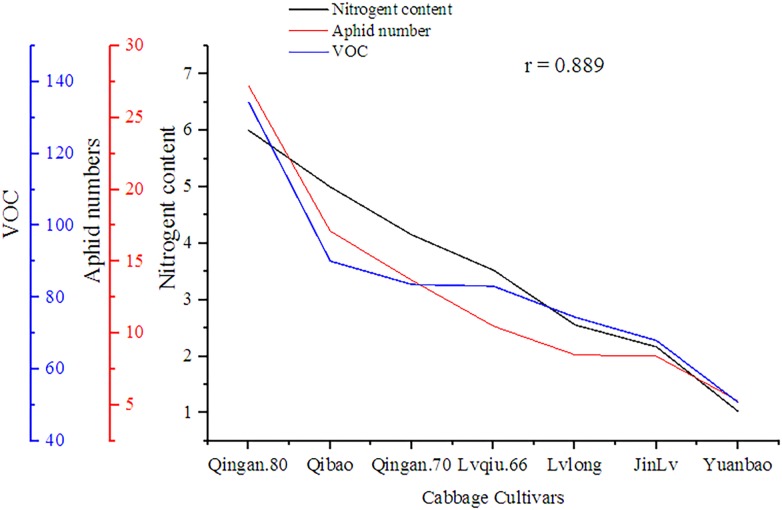
Correlation of nitrogen content, VOC, with wingless *M. persicae.*

A significant (*P* = 0.002) and positive (*r* = 0.858) correlation was observed between wingless *M. persicae*, VOCs and nitrogen content among cabbage cultivars. As the nitrogen increased, VOCs also increased, similarly, the aphid preference also increased (Figure 9).

## Discussion

The behavioral response of herbivore can be altered based on their preference for host plant cultivars. In this study, *M. persicae* was attracted differently among seven cabbage cultivars, providing new insight into the relationship between herbivore host plant selections based on semiochemicals. These semiochemicals can potentially be used in pest control by manipulating the behavior of *M. persicae*. The results showed that wingless *M. persicae* exhibited a significant preference to the odors of Qingan 80 but no attraction to the odors of Yuanbao plants. In the olfactometer experiments, herbivore attraction to the host plants mainly depend on the plant olfactory cues. Therefore, we assume that plant volatiles play a pivotal role for *M. persicae* host plant selection. Our results was supported by a previous study showing different behavioral preference of *M. persicae* on odor blends of four potato cultivars (Eigenbrode et al., 2002; Werner et al., 2009; Rajabaskar et al., 2013). Although it is not known yet which VOCs are responsible for the attraction of *M. persicae*, some VOC blends may act as kairomones for herbivorous insects (Park and Hardie, 2003; Park et al., 2005; Tasin et al., 2007; Najar-Rodriguez et al., 2010; Webster et al., 2010).

Plant cultivars can be categorized based on the emitted VOCs, which indicate a remarkable correlation between herbivore attraction and mixture of volatile emission (Bleeker et al., 2009; Darshanee et al., 2017). Our PCA results separated the most attractive, the most repellent and moderate attractive plants based on the VOCs. These results imply a consistent relationship between volatile chemical compounds emitted by the host plants and the preference behavior of *M. persicae*. It has been recognized that aphids use their antennae to detect odor emitted by their host plants, which could influence their acceptance of host plants (Picket et al., 1992; Storer et al., 1996). The host plant searching behavior of the bean black aphid, *Aphis fabae* Scopoli, was affected by15 chemical compounds (Webster et al., 2008a,b; Webster et al., 2012), while the wingless aphids responded to 11 different compounds (Kasmi et al., 2017). We have identified 19 important chemical compounds that showed significant differences among the cabbage cultivars, but 13 compounds showed significant differences among highly attractive and repellent cultivars.

The majority of volatiles emitted from the cabbage cultivars belong to terpene and GLVs classes. Most terpenes are info chemicals for herbivore species when emitted from the related host plants (Francis et al., 2005). Since aphids are known to discriminate small changes in the ratios of volatile compounds (Webster et al., 2012), the overall blend of volatiles emitted is likely responsible for host selection of *M. persicae* (Ngumbi et al., 2007). We found that the higher amount of total headspace volatile emission of Qingan 80 compared with the repellent cultivar Yuanbao, which may help to explain the strong arresting behavior of the aphids. However, it remains possible that the aphid behavior does not rely on particular compounds, but rather specific combinations and that many different blends can be attractive for *M. persicae*. Indeed, aphids often show strong responses to a blend of VOCs compared with an individual VOC (Michael et al., 2010; Bruce and Pickett, 2011). The behavior of the aphids can be altered by removing any one component or VOC class (Ngumbi et al., 2007). Some volatile substances, such as (Z)-3-hexen-1-ol, α-pinene, (E)-β-caryophyllene, α-humulene and azulene, are related to host plant selection of herbivore pests (Williams et al., 2005; Domingue et al., 2016; Darshanee et al., 2017). These results are consistent with our finding that the VOCs detected were generally the same as detected previous studies. In our study, α-caryophyllene, α-phellendrene, hexanal and *Trans-*2-hexanal were prominent chemical compounds in the most aphid attractive cultivar Qingan 80. The emission levels of α-caryophyllene within volatile blends can repel or attract herbivores and enhance recruitment of biological control agents (Cook et al., 2007; Kos et al., 2013). α-phellandrene has also been shown to have similar chemical properties that can act as herbivore attractive chemical compounds (Raffaele et al., 2007; Kreuzwieser et al., 2014). Moreover, some research evidence has shown the ability to change behavioral responses of herbivorous when plants emit more hexanal and *Trans-*2-hexanal chemical constituents (Rodriguez-Saona et al., 2006; Von Merey et al., 2011). However, (E)-β-farnesene and *cis-*α-farnesene emission was considerably higher in the cultivar Yuanbao which was least preference cultivar for the aphids. (E)-β-farnesene elicited a negative behavioral response toward aphids, which suggests it might be a repellent and is considered to be the main component of alarm pheromones released by aphids in response to parasitoids or predator attacks (Hardie et al., 1999; Verheggen et al., 2007; Vandermoten et al., 2012). (E)-β-farnesene is released from the specialized foliar trichomes in numerous plant species and is a common element of volatile blends induced by herbivore (Szafranek et al., 1998; Cavaleiro et al., 2002; Turlings and Ton, 2006).

Plant volatile emission and nutrients have a relationship. Among the plant nutrients, nitrogen is frequently considered as a limiting resource for insects, and the nitrogen content in plant leaves is used as a food quality indicator, as well as an important factor in host selection by phytophagous insects (Scriber and Slansky, 1981). We found that *M. persicae* performed better on plants with higher nitrogen than those with lower nitrogen content. This trend was more obvious in some cultivars. Therefore, we hypothesized that high or low nitrogen affected the performance of the aphids differently. For instance, Yuanbao, which had low nitrogen, was more resistant to the aphids, but Qingan80, which contained high amount of nitrogen, was more susceptible to aphids. Similar relationships have been commonly found in the studies dealing with plant nutrition (Ponder et al., 2000; Lawlor et al., 2001; TerSteege et al., 2001; Liu et al., 2016). The nitrogen content of host plants is significantly correlated with herbivore attraction and development time (Jallow and Zalucki, 2003; Van Tol et al., 2004; Storey, 2007). This has been shown by host preference, offspring performance and intrinsic rate of increase (*r*_m_) of aphids. In the current study, we showed a remarkable relationship among *M. persicae* attraction, plant nitrogen content and total volatile emissions. It implies that nitrogen content of the plants has a significant impact on volatile emission and preference behavior of *M. persicae.* In line with our findings, some research evidence showed that the nitrogen level on cotton plants directly affected plant volatile emissions, as well as attraction of the herbivore pest, *Spodoptera exigua* (Olson et al., 2009). In future studies, it will be necessary to examine the attraction level of parasites or predators for the host plants, which were more abundant than the *M. persicae*, as there might be correlation among natural enemy attraction, plant volatile emission and nitrogen content in cabbage plants.

## Conclusion

This experiment provide a positive relationship between nitrogen content and VOC emission in leaves of cabbage cultivars. Our finding regarding the interaction between *M. persicae* attraction, nitrogen content and VOC emissions might be exploited as an important component for IPM programs, although volatile chemical application is currently in its infancy for practical usage under open field conditions. Future studies should focus on investigating the role of particular compounds emitted after herbivore damage and identifying changes in the overall emitted volatile blends, which would significantly enhance our understanding of the olfactory cues used in host plant selection behavior of aphids.

## Author Contributions

NA, HLCD, and T-XL conceived and designed the experiments and wrote the manuscript. NA, HLCD, and Z-FZ performed the experiments. NA, HLCD, and IK analyzed the data.

## Conflict of Interest Statement

The authors declare that the research was conducted in the absence of any commercial or financial relationships that could be construed as a potential conflict of interest.
